# Impact of parental history of substance use disorders on the clinical course of anxiety disorders

**DOI:** 10.1186/1747-597X-2-13

**Published:** 2007-04-27

**Authors:** Maria E Pagano, Richard Rende, Benjamin F Rodriguez, Eric L Hargraves, Amanda T Moskowitz, Martin B Keller

**Affiliations:** 1 Department of Psychiatry, Division of Child Psychiatry, Case Western Reserve University, Euclid Avenue, Cleveland, OH, USA; 2 Department of Psychiatry and Human Behavior, Brown University, Butler Hospital, Blackstone Boulevard, Providence, RI, USA; 3 Department of Psychology, Southern Illinois University, Lincoln Drive, Carbondale, IL, USA

## Abstract

**Background:**

Among the psychological difficulties seen in children of parents with substance use problems, the anxiety disorders are among the most chronic conditions. Although children of alcoholic parents often struggle with the effects of parental substance use problems long into adulthood, empirical investigations of the influence of parental substance use disorders on the course of anxiety disorders in adult offspring are rare. The purpose of this study was to examine prospectively the relationship between parental substance use disorders and the course of anxiety disorders in adulthood over the course of 12 years.

**Methods:**

Data on 618 subjects were derived from the Harvard/Brown Anxiety Research Project (HARP), a longitudinal naturalistic investigation of the clinical course of multiple anxiety disorders. Kaplan-Meier survival estimates were used to calculate probabilities of time to anxiety disorder remission and relapse. Proportional hazards regressions were conducted to determine whether the likelihood of remission and relapse for specific anxiety disorders was lower for those who had a history of parental substance use disorders than for individuals without this parental history.

**Results:**

Adults with a history of parental substance use disorders were significantly more likely to be divorced and to have a high school level of education. History of parental substance use disorder was a significant predictor of relapse of social phobia and panic disorders.

**Conclusion:**

These findings provide compelling evidence that adult children of parents with substance use disorders are more likely to have relapses of social phobia and panic disorders. Clinicians who treat adults with anxiety disorders should assess parental substance use disorders and dependence histories. Such information may facilitate treatment planning with regards to their patients' level of vulnerability to perceive scrutiny by others in social situations, and ability to maintain a long-term panic-free state.

## Background

A substantial literature currently exists showing that children from families with parental substance use disorders are at greater risk for the development of psychiatric and psychosocial difficulties. Children and adolescents who have parents with substance use disorders have been shown to be at greater risk for developing drug and alcohol abuse problems of their own, as well as problems with anxiety and depression, low self-esteem, poor relationships, and poor global functioning [1-7]. In adulthood, offspring of parents with substance use disorders are generally viewed as at increased risk for lower global functioning, lower educational attainment, more marital difficulties, and greater likelihood of psychiatric disorders than their peers with parents who do not have substance use disorders [8-10].

Among the psychological difficulties seen in children of parents with substance use problems, the anxiety disorders are among the most chronic conditions. Prior investigations have shown the various anxiety disorders to have a generally chronic course, with few sufferers achieving a completely symptom free state for extended periods of time [11-13]. To date, however, few investigations have been conducted that directly compare the phenomenology of the anxiety disorders among individuals who did and did not have parents with substance abuse problems. Based on preliminary evidence, there is reason to believe anxiety disorder patients with parents who were substance abusers may differ from anxiety disorder patients without such parents. In the Epidemiological Catchment Area (ECA) study, for example, significantly higher lifetime rates of specific anxiety disorders were found in 407 adult children of alcoholics compared to 1,477 matched control subjects who did not have alcoholic parents: generalized anxiety disorder (15% versus 8%), panic disorder (2.7% versus 0.7%), agoraphobia (8.6% versus 4.9%), and social phobia (3.7% versus 2%)[14]. However, the majority of these differences were not found when comparing current rates of these anxiety disorders. Given the chronicity of these disorders, it is not clear the extent to adult children of alcoholics/addicts are more susceptible to endure a prolonged length of time until remission from an anxiety episode or are more vulnerable to reoccurrence of an anxiety episode. Achieving a better understanding of how parental substance use disorders affect the clinical course of anxiety may aid treatment providers with diagnosing and treating anxiety disorders.

The few studies that have attempted to examine the relationship between parental substance use disorders and the patterns and longitudinal course of anxiety disorders in adulthood suffer from several noteworthy methodological limitations. First, the great majority of studies utilize retrospective, cross-sectional designs, with, at most, one or two follow-up time points [4]. Such study designs do not adequately take into account the empirically demonstrated chronicity of anxiety disorders and are weaker than prospective longitudinal studies. For example, if the rate of change in anxiety symptomatology (i.e., reducing or increasing of anxiety symptoms) is affected by having a history of parental substance use disorders, a study with a limited number of follow-ups would fail to capture adequately such variation. Similarly, no study to date has examined specifically the impact of parental substance use disorders on the clinical course of one, let alone multiple types of anxiety disorders in offspring across the lifespan developmental stage of adulthood.

Clinical comorbidity and functional impairment have also not sufficiently been addressed in prior studies examining the course of anxiety disorders in patients with and without parents with substance abuse problems. Diagnostic comorbidity with other anxiety disorders, major depression, and substance use disorders is quite common [15,16] and has been associated with a poorer course of anxiety conditions [11,17]. Similarly, poorer general functioning has been associated with a reduced probability of remission and a greater probability of relapse for anxiety disorders [18]. Prior longitudinal examinations have shown that more diagnostic comorbidity and poorer psychosocial functioning [12,13] are associated with a more chronic course of anxiety symptomotology. Moreover, patients with anxiety disorders are at increased risk for diagnosis with multiple anxiety, depressive, and other conditions [16]. Finally, few studies have examined other confounding variables like childhood abuse, parental anxiety problems, and the development of substance abuse disorders in offspring themselves. Only one study to date has controlled for childhood abuse. In a study of 90 families, parental alcoholism and sexual abuse were found to be independent predictors of increased interpersonal anxiety among youth [19].

The goal of the present study is to examine how family history of substance use disorders affects the clinical course of anxiety disorders in adult offspring by providing appropriate attention to prior methodological weaknesses. The current study examines the impact of parental substance use disorders on the longitudinal course of anxiety disorder symptoms in the largest sample of adults currently being prospectively followed for anxiety disorders. These examinations include the observation of multiple types of anxiety disorders over significant lengths of time. In addition, we utilized family history assessments that include both parental drug and alcohol abuse/dependence, and assessed the potential contribution of childhood abuse and parental anxiety. This study addresses three basic questions: (1) Is the course of anxiety disorders in adulthood impacted by a history of parental substance use disorders? (2) What specific anxiety disorders are most affected by parental substance use disorders? (3) How are these patterns altered when childhood abuse, parental history of anxiety disorders, and diagnostic comorbidity, and functional impairment are taken into account?

## Methods

This study was conducted using data from the Harvard/Brown Anxiety Research Project (HARP), a longitudinal, prospective, short-interval follow-up study of adults with a current or past history of anxiety disorders. From 1989–1991, 711 participants entered the HARP study from over 30 clinicians' practices at 11 different clinical treatment facilities in the New England area. These methods are described in detail elsewhere [20]. Inclusion criteria included a past or current diagnosis of the following at intake: panic disorder without agoraphobia (PD), panic disorder with agoraphobia (PDA), social phobia (SP), or generalized anxiety disorder (GAD). Insufficient for inclusion, but frequently seen as comorbid conditions, were diagnoses of simple phobia, posttraumatic stress disorder, obsessive-compulsive disorder, or anxiety disorder not otherwise specified. Participants must have been at least 18 years of age at intake, willing to voluntarily participate in the study, and sign a written consent form. Exclusion criteria consisted of the presence of an organic brain syndrome or a history of schizophrenia, or current psychosis at intake. Any other comorbidity was included. Because the focus of this study is the course of anxiety disorders, this study excludes 5% of the 711 participants in the original sample who were interviewed at intake only. There were no significant differences in the distribution of anxiety disorder diagnoses, illness severity, demographic characteristics including gender, age of episode onset, and clinical correlates of subjects in the study sample and those without follow-up data.

### Measures

#### Diagnostic assessment

The initial comprehensive evaluation assesses lifetime history using the Structured Clinical Interview for DSM-III-R Non-Affective Disorders, Patient Version (SCID-P) and the Research Diagnostic Criteria (RDC) Schedule for Affective Disorders-Lifetime (SADS-L). Items on the SCID-P and SADS-L were combined to create the SCALUP, a structured interview used to assess diagnoses at intake (available from M.B. Keller upon request). The instrument yielded both present and past RDC diagnoses for affective disorders and DSM-III-R diagnoses for nonaffective (including anxiety) disorders. Interviews, conducted by trained, experienced clinical interviewers whose educations ranged from B.A. to M.A., usually took place in single sessions lasting 2 to 4 hours.

Follow-up interviews were conducted at 6-month intervals for the first two years, annually for Years 3–6, and every six months in Years 7–12. These interviews utilized the Longitudinal Interval Follow-up Evaluation-Upjohn (LIFE-UP, [21]), which assesses the weekly course of Axis 1 disorders with psychiatric status ratings (PSR's; Table 1). The greatest severity of illness, a PSR 6, requires full DSM-III-R criteria in addition to severely disrupted functioning. A PSR of 1 indicates an absence of symptoms.

**Table 1:** Weekly Psychiatric Status Rating Scale

Code	Term	Criteria
6	Full Criteria, Severe	Meets DSM-III-R or RDC criteria for definite criteria, severe symptoms, or extreme impairment in functioning
5	Full Criteria	Meets criteria for definite symptoms but no extreme impairment in functioning
4	Marked	Does not meet criteria but has major symptoms of impairment from this disorder
3	Partial Recovery	Considerable less psychopathologic impairment than full criteria with no more than moderate impairment in functioning, but still has obvious evidence of disorder
2	Residual	Either patient claims not to be completely his/her self, or rater notes presence of symptoms in this disorder in no more than mild degree
1	Usual Self	Patient is returned to usual self without any residual symptoms of this disorder, but may have significant symptoms from some other condition or disorder; if so, this should be recorded under that condition or disorder

Reliability and validity studies conducted on the LIFE found good to excellent agreement on PSR scores. Intraclass correlation coefficients (ICCs) for each of the disorders were as follows: 0.67–0.88 for panic disorder, 0.78–0.86 for generalized anxiety disorder, 0.75–0.86 for social phobia, and 0.73–0.74 for major depressive disorder [22]. Long term test-retest over one year also found very good to excellent reliability for the anxiety disorders and for major depressive disorder. A separate external validity assessment comparing PSR's with other psychosocial measures found good concurrent and discriminant validity [22].

#### History of parental substance use and anxiety disorders

History of parental SU and anxiety disorders were assessed with the Family-History Research Diagnostic Criteria (FH-RDC; [23]). The FH-RDC is an interviewer-administered measure that assesses the history of psychiatric disorders of immediate family members identified by the respondent. For the presence of alcoholism and drug use disorder, the FH-RDC uses the RDC criteria for both substances. Two criteria are required to establish the diagnosis: 1) problem with substance use not limited to one isolated incident and 2) at least one substance use related problem with legal problems, health problems, marital or family problems, work problems or impairment as housekeeper, treatment for substance use, or social problems, such as fights or loss of friends. The FH-RDC has been shown to accurately reflect the psychiatric history of family members of the respondent [24]. Interrater reliability for chance corrected agreement (Kappa) for the diagnosis of parental alcoholism and drug use disorders has been shown to be excellent [25]. Test-retest interrater reliability for specific anxiety disorders has been shown to be good-excellent [26].

#### Trauma

Interviewers recorded all traumatic events reported by subjects in response to the SCID PTSD trauma probe. Subjects were asked if they experienced the traumatic events of sexual abuse or physical abuse during childhood. The full HARP sample fell intro three categories: those who met full criteria for PTSD, subjects who had trauma histories but did not meet criteria for PTSD, and subjects without a history of trauma. These methods are described in detail elsewhere [27].

#### Definitions of remission and relapse

Remission and relapse in this study were defined prospectively. Participants were considered to have recovered from his or her anxiety disorder if he or she experienced eight consecutive weeks at PSR ratings of 2 or less (see Table 1). A subject who met this condition was virtually asymptomatic for two consecutive months. This definition of remission has been widely used in studies of affective and other disorders. Relapse was defined (with the exception of GAD) as the onset of symptoms at a PSR level of 5 or greater for two consecutive weeks following a remission (see Table 1). For GAD, relapse was defined as the onset of symptoms at a PSR 5 or greater for 6 months following a remission.

### Statistical analysis

Statistical analyses were conducted using SAS version 8.0 [28], using PROC FREQ, PROC ANOVA, PROC LIFETEST, and PROC PHREG. Depending on the type of variables (continuous or discrete), analysis of variance (ANOVA) or chi-square analyses were performed to evaluate demographic and clinical differences between groups. Kaplan-Meier survival estimates were used to calculate probabilities of time to remission and time to relapse of anxiety disorders. Cox proportional hazards regressions were conducted to determine whether the likelihood of remission and relapse was more adverse for subjects with a history of parental substance use disorders. Survival analysis and cox proportional hazard regression analysis make no assumptions about underlying survival distributions and allow for the examination of prognostic factors when estimating in time to occurrence of an event [30]. To identify the unique value of parental SU on the course of each anxiety disorder, the potential confounds of childhood physical and sexual abuse, total number of comorbid anxiety disorders, parental anxiety disorder, and the course of SU in offspring themselves were entered into cox regression models as covariates. Non-significant covariates (p > .50) were removed from cox regression models using stepwise approaches for cox regressions [30].

## Results

### Sample demographic and baseline clinical characteristics

Sample demographic and clinical characteristics are described in Table 2. Study participants reported a psychiatric history for 580 mothers and 191 fathers. A total of 111 (18%) subjects reported a history of parental SU (92 mothers and 24 fathers). Of mothers with SU, 68% reported alcohol abuse/dependence, 15% reported drug abuse/dependence, and 16% reported both. These percentages were similar among fathers with SU: 63% reported alcohol abuse/dependence, 20% reported drug abuse/dependence, and 17% reported both. On average, subjects were 17 years of age at the onset of parental SU (M = 17.39, SD = 10.36). A total of 145 (23%) participants had a history of a parental anxiety disorder (119 mothers and 32 fathers). At intake, the HARP study sample ranged in age from 18 to 86 years (M = 41.02, SD = 12.53). Females were predominant 2:1 over males. Minorities represented only 3% of the sample, reflecting the patient populations of the recruiting sites. The majority (53%) was married, 39% had completed college or graduate school and 46% of subjects were working full-time. In terms of clinical characteristics, 148 (24%) of the study sample were in episode of GAD at intake, 151 (24%) SP, 311 (50%) PDA, and 72 (12%) PD. A total of 53 subjects (9%) reported a childhood history of sexual abuse and 38 (6%) reported a childhood history of physical abuse.

**Table 2:** Demographics and Clinical Characteristics of Sample

	Total N = 618(100%)	No Parental SU^1^ N = 507 (82%)	Parental SU^1^ N = 111 (18%)
Demographic Characteristics			
Female (%)	413 (67%)	338 (67%)	75 (68%)
			
Work full-time (%)	285 (46%)	235 (46%)	75 (68%)
			
Marital Status (%)			
Married	328 (53%)	268 (53%)	60 (54%)
Live in Partner	32 (5%)	24 (5%)	8 (7%)
Divorced	93 (15%)	70 (14%)	23 (21%)†*
Never Partner	165 (27%)	145 (29%)	20 (18%)
			
Education (%)			
College grad or more	243 (39%)	222 (44%)	21 (19%)†***
Partial college/HS	329 (53%)	250 (49%)	79 (71%)
Less than HS	46 (8%)	35 (7%)	11 (10%)
			
Age (M, SD)	41.02 (12.53)	42.89 (12.48)	40.61 (12.51)
			
Clinical Characteristics^6^			
GAD^2 ^(%)	148 (24%)	123 (24%)	25 (23%)
Age of GAD onset (M, SD)	21.50 (14.45)	21.04 (14.50)	23.80 (14.31)
			
SP^3 ^(%)	151 (24%)	123 (24%)	28 (25%)
Age of SP onset (M, SD)	14.81 (8.51)	14.77 (8.72)	15.01 (7.65)
			
PDA^5 ^(%)	311 (50%)	253 (41%)	58 (52%)
Age of PDA onset (M, SD)	26.93 (10.99)	26.61 (10.89)	28.31 (11.42)
			
PD^4 ^(%)	72 (12%)	59 (12%)	13 (12%)
Age of PD onset (M, SD)	34.78 (12.10)	34.55 (12.09)	35.82 (12.60)
			
Childhood sexual abuse	53 (9%)	39 (8%)	14 (13%)
Childhood physical abuse	38 (6%)	27 (4%)	11 (10%)

^1^Substance Use Disorder, ^2^GAD = Generalized Anxiety Disorder, ^3^SP = Social

^4^PDA = Panic Disorder without Agoraphobia, ^5^PDA = Panic Disorder with Agoraphobia, ^6^Totals exceed 100% because of comorbidity

† Chi-Square test

* p < .05 *** p < .0001

As shown in Table 2, there were no significant differences between subjects with and without parental SU in terms of gender, status of full-time work, age, diagnostic clinical characteristics, and childhood history of abuse. However, subjects with parental SU were significant more likely to be divorced (*χ*^2 ^= 7.81, *df*= 3, *p*< 0.05), and to have completed only a high school level of education (*χ*^2 ^= 23.60, *df*= 2, *p*< 0.0001). Subjects with a history of parental SU were significantly more likely to have a personal history of SU in comparison to subjects without a history of parental SU (*χ*^2 ^= 21.45, *df*= 1, *p *< 0.0001).

### 12-year longitudinal course

#### Remission

With the exception of subjects with panic disorder without agoraphobia (PD), a majority of subjects still maintained the same anxiety disorder diagnosis they had 12 years after study entry. Using Kaplan-Meier survival estimates, subjects with PD were more likely to have a remission at all time points and had a 0.82 probability of achieving remission from their intake episode of PD by year 12. In contrast, patients with panic disorder with agoraphobia (PDA), generalized anxiety disorder (GAD), and social phobia (SP) had much lower probabilities of achieving remission over 12 years of follow-up. Patients with GAD had a 0.58 probability of achieving remission, while those with PDA had a 0.48 probability of recovering from their intake episode over 12 years of follow-up. Patients with social phobia had the least probability of recovering after 12 years (0.37).

#### Relapse

Subjects who remitted from their intake anxiety disorder had a high probability of subsequently having a relapse over the follow-up period. Although subjects with PD were found to have a higher likelihood of remission compared to subjects with PDA, the probability of relapse was similar (0.56 vs. 0.58 respectively). Individuals with GAD or SP who recovered were somewhat more likely to have a relapse over the 12 year follow-up period. The probability of having a relapse of social phobia was 0.39, while the probability of relapse in patients who had recovered from GAD was 0.45 at the end of the 12 years.

### Predicting anxiety course based on parental history of substance use disorder

Next, we used cox proportional hazard regression analyses to evaluate whether parental SU predicted remission from, and relapse to, GAD, SP, PD, and PDA. Demographic characteristics (gender, marital status, education, age), childhood sexual or physical abuse, and the number of comorbid anxiety disorders were not significant predictors of the course of anxiety disorders in the study sample. History of parental anxiety did not reach significance as a predictor for remission or relapse of anxiety disorders. The course of personal SU was significant only as a predictor of GAD relapse (a full report of the non-significant findings are available upon request). Thus, cox proportional hazard regression models were reported without demographic or child abuse variables, but controlled for covariates below the significance threshold of p < .50 (history of parental anxiety, course of personal SU, anxiety disorder comorbidity). There was no violation of the proportional hazards assumption in cox regression models.

**Figure 1:**
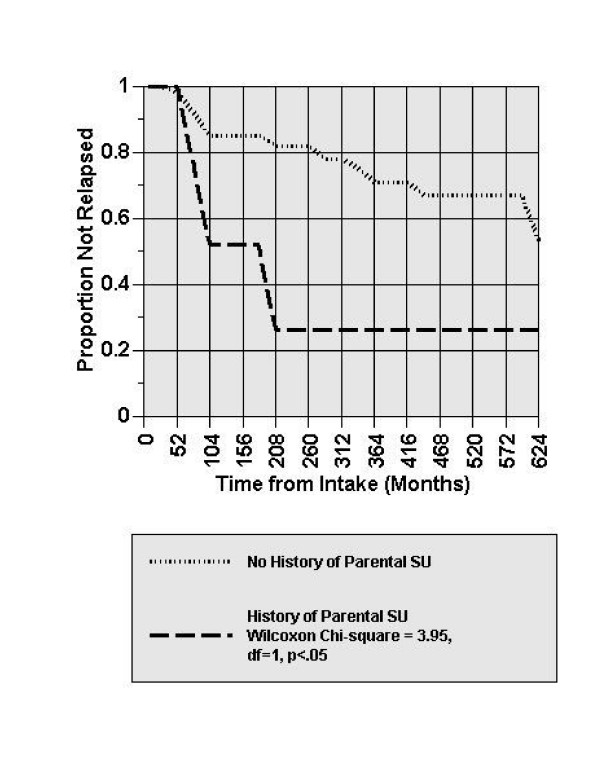
**Relapse Course of Social Phobia Disorder by History of Parental SU**. Figure 1 illustrates the course of SP relapse over 12 years by those with and without a history of parental SU. The likelihood of SP relapse was significantly greater for those with a history of parental SU.

As shown in Table 3, history of parental SU was not a significant predictor of remission for any of the studied anxiety disorders. However, history of parental SU significantly predicted relapse in two of the studied anxiety disorders: SP and PD. Results of the cox proportional hazard regression analyses showed that individuals with a history of parental SU were approximately four times more likely to have a SP relapse compared to those without a history of parental SU (Wald X^2 ^= 6.27, *p *< .05, HR = 4.12). Figure 1 illustrates the course of SP relapse over 12 years by those with and without a history of parental SU. The probability of having a SP relapse was 0.74 for adults with a history of parental SU in comparison to .47 for those with no history of parental SU. Similarly, those with a history of parental SU were significantly more likely to have a PD relapse (Wald X^2^= 5.54, *p *< .05, HR = 3.39). The probability of having a PD relapse was .78 for those with a history of parental SU in comparison to .52 for those with no history of parental SU. No adults with parental SU occurred both SP and PD relapses.

**Table 3:** Cox Proportional Hazard Regressions Predicting Anxiety Disorder Remission and Relapse by History of Parental SU^1^

Remission							
	Eligible	Remit	Chi-Square	p	Hazard Ratio	95% CI	
SP	147	46	2.12	0.14	0.49	0.19	1.26
GAD	148	75	0.45	0.50	0.79	0.47	1.43
PDA	311	144	0.03	0.85	1.01	0.67	1.54
PD	67	47	0.32	0.57	1.25	0.58	2.66
							
Relapse							
	Eligible	Relapse	Chi-Square	p	Hazard Ratio	95% CI	
SP	46	16	6.27	<.05	4.12	1.08	12.82
GAD	75	35	1.55	0.21	0.46	0.14	1.56
PDA	144	65	2.36	0.12	1.59	0.88	2.87
PD	47	20	5.54	<.05	3.39	1.23	9.38

^1^SU = Substance Use Disorder, ^2^GAD = Generalized Anxiety Disorder, ^3^SP = Social

^4^PDA = Panic Disorder without Agoraphobia, ^5^PDA = Panic Disorder with Agoraphobia

## Discussion

After controlling for childhood physical and sexual abuse as well as history of parental anxiety disorders, the course of SU in participants themselves, and anxiety disorder comorbidity, we found a four-fold increase in risk for the relapse of social phobia among adults with a history of parental substance use disorders. In contrast to the other anxiety disorders, the onset date of social phobia was during adolescence, and thus may be more likely to show a stronger influence of parental behavior in adulthood. Individuals in families with substance use disorders tend to employ more and more isolation as a defense, minimizing the amount of potential embarrassment to which they are exposed [29]. These results suggest an important relationship between the extent to which parents exhibit substance abuse problems and an individual's level of vulnerability to perceive scrutiny by others in social situations in adulthood.

Similarly, adults with a history of parental substance use disorders were more likely to have a PD relapse following a period of remission. This is an interesting finding suggesting that a history of parental substance use disorders negatively impacts an adult's ability to maintain long-term panic-free state. Moreover, it should be noted that panic attacks, which are the hallmark feature of PD, are complicated and variable phenomena that involve both physical symptoms (i.e. heart palpitations, paresthesias, chest pain) and cognitive symptoms (i.e. fear of losing control, depersonalization), It could not be determined in the current study if a history of parental substance use had a differential effect on the variety of panic symptoms patients experience or the frequency that certain symptoms recur. An interesting avenue of future research would be to explore the differential course of specific panic symptoms as a function of the presence or absence parental substance use disorders.

Results from this study are the first to present a lifespan perspective of the potential impact of parental substance use disorder on the course of anxiety disorders in adulthood. Like other researchers who have used similar methodologies to examine psychiatric illnesses presenting in adult children of alcoholics/addicts [7], the present study has several methodological strengths that allow a more extensive examination of the role of a history of parental SU on the course of anxiety in adulthood. HARP has prospectively followed a large sample of subjects with anxiety disorders for over a decade, using comprehensive follow-up assessments that gathered information on psychiatric disorders in short-interval follow-ups. The naturalistic design of this study offers a real world perspective on the course of anxiety illness in clinical populations. In addition, HARP examines the prevalence of anxiety disorders in general and not specifically regarding family history of substance use disorders. Therefore, the results of the current investigation may be less likely to be influenced by false positives of parental substance use disorders.

This study does have some limitations that need to be considered. First, the study uses a clinical sample that was treatment-seeking when originally recruited. As such, the results may not be generalizable to non-clinical and/or non-treatment-seeking populations of anxiety patients. Second, our assessments were also limited by a lack of direct assessment of the status of parental substance use disorders, relying instead on patient self-report of their parents' behaviors. Given the tendency for children of alcoholics/addicts to underreport parental SU, the findings observed may be conservative. Third, although approximately one-third of parents with SU had drug use disorders, the limited number of SU parents with only drug use disorders precluded examination of the independent effects of parental drug use disorders. Fourth, the number of events (remissions or relapses) limited the number of apriori predictor variables to be tested with adequate power in cox regression models. Future investigations are warranted that examine important environmental and personal characteristics that may negate or exacerbate the effects of parental SU. Finally, the sample was primarily Caucasian and thus generalizing our results to non-Caucasian populations is not advisable.

## Conclusion

The long-term influence of parental substance use disorders on the course of anxiety disorders among children of alcoholics/addicts and beneficial treatments specific to these individual are potent areas for future work. An informed clinical policy in favor of assessment of parental substance abuse/dependence histories in the treatment of anxiety disorders may be particularly appropriate for individuals with social phobia. These individuals may benefit from long-term maintenance therapy that includes pharmacological and/or non-pharmacological approaches. The findings from this prospective longitudinal investigation highlight the importance of studies that examine the time course of change in anxiety diagnostic status in relation to parental history of substance use disorders.

## Competing interests

The author(s) declare that they have no competing interests.

## Authors' contributions

MP conceived of the study, participated in its design, conducted all statistical analyses, and helped to draft the manuscript.

RR conceived of the study and helped to draft the manuscript.

BR participated in the design of the study and helped to draft the manuscript.

EH participated in the design of the study and helped to draft the manuscript.

MK conceived of the study and helped to draft the manuscript.

AM assisted in the revision of the final manuscript.

All authors read and approved the final manuscript.
